# Galacto-Oligosaccharide Alleviates Alcohol-Induced Liver Injury by Inhibiting Oxidative Stress and Inflammation

**DOI:** 10.3390/metabo12090867

**Published:** 2022-09-15

**Authors:** Shipeng Zhou, Qiuhua Tan, Bingjian Wen, Yan Bai, Qishi Che, Hua Cao, Jiao Guo, Zhengquan Su

**Affiliations:** 1 Guangdong Engineering Research Center of Natural Products and New Drugs, Guangdong Provincial University Engineering Technology Research Center of Natural Products and Drugs, Guangdong Pharmaceutical University, Guangzhou 510006, China; 2 Guangdong Metabolic Disease Research Center of Integrated Chinese and Western Medicine, Key Laboratory of Glucolipid Metabolic Disorder, Ministry of Education of China, Guangdong TCM Key Laboratory for Metabolic Diseases, Guangdong Pharmaceutical University, Guangzhou 510006, China; 3 School of Public Health, Guangdong Pharmaceutical University, Guangzhou 510310, China; 4 Guangzhou Rainhome Pharm & Tech Co., Ltd., Science City, Guangzhou 510663, China; 5 School of Chemistry and Chemical Engineering, Guangdong Pharmaceutical University, Zhongshan 528458, China

**Keywords:** alcoholic liver disease (ALD), antioxidant, anti-inflammatory, galacto-oligosaccharide, hepatoprotective

## Abstract

Alcoholic liver disease (ALD) is a primary cause of mortality and morbidity worldwide. Oxidative stress and inflammation are important pathogenic factors contributing to ALD. We investigated the protective mechanism of galacto-oligosaccharide (GOS) against ALD through their antioxidant and anti-inflammatory activities by performing in vivo and in vitro experiments. Western blot and RT‒PCR results indicated that the expression of cytochrome P450 protein 2E1 (CYP2E1) in liver tissues and L02 cells was reduced in the GOS-treated mice compared with the model group. In addition, GOS prominently reduced the expression of Kelch-like ECH-associated protein 1 (Keap1), increased the expression of the nuclear factor erythroid-2-related factor 2 (Nrf2) and haem oxygenase-1 (HO-1) proteins, and enhanced the antioxidant capacity. In addition, GOS decreased inflammation by reducing inflammatory factor levels and inhibiting the mitogen-activated protein kinase (MAPK)/nuclear factor kappa B (NF-κB) pathway. Based on these results, GOS may be a prospective functional food for the prevention and treatment of ALD.

## 1. Introduction

Alcoholic liver disease (ALD) caused by long-term excessive alcohol intake is the main cause of alcohol-related mortality and morbidity. It is a major disease burden worldwide and has become one of the problems affecting global health [1]. ALD involves multiple pathological stages caused by acetaldehyde and persistent cell poisoning, ranging from initial liver steatosis to more advanced forms, such as alcoholic hepatitis, liver fibrosis, cirrhosis, and even liver cancer, which seriously threaten human health [2,3,4]. Currently available drugs for the treatment of ALD include corticosteroids and pentoxifylline, but the effect remains unsatisfactory [5]. However, no drugs have been approved by the Food and Drug Administration (FDA) to treat ALD [6]. Therefore, the exploration of healthy and safe functional foods is a promising direction to prevent and alleviate ALD.

For more than 50 years, alcohol has been considered a direct liver toxin. Alcohol is primarily metabolized in the liver, with more than 95% of alcohol metabolized in this organ and only 2~5% directly excreted through urine, sweat, and exhalation [7]. When an individual consumes alcohol, CYP2E1 is the key liver enzyme involved in alcohol metabolism. Ethanol is metabolized by alcohol dehydrogenase (ADH) to produce acetaldehyde. CYP2E1 becomes involved in metabolizing ethanol to acetaldehyde at elevated ethanol concentrations. Acetaldehyde is further metabolized to acetate by aldehyde dehydrogenase 2 (ALDH2). The activity of ADH and ALDH2 results in the formation of NADH and increases the ratio of reduced NADH to the oxidized NAD^+^. Ethanol metabolism by CYP2E1 and the reoxidation of NADH via the electron transport chain in the mitochondria both lead to large amounts of rapidly produced ROS, causing oxidative stress. High levels of ROS exacerbate the inflammatory response, which then releases large amounts of pro-inflammatory cytokines such as TNF-α, IL-1, and IL-6. It also activates NF-κB and MAPK pro-inflammatory pathways, thereby enhancing the inflammatory response involving inflammatory cell infiltration, cell necrosis, and apoptosis [8]. Therefore, the inhibition of oxidative stress and inflammation is a promising direction for the treatment of ALD.

**Figure 1:**
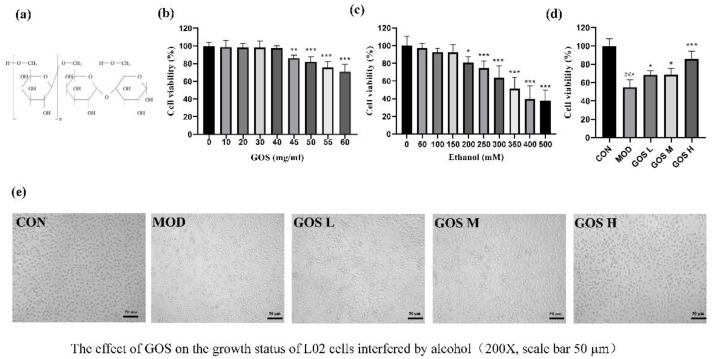
Chemical structure of GOS, screening of GOS administration concentration and alcohol-modeling concentration, effects of GOS on the viability of L02 cells treated with ethanol, and effects of GOS on the growth status of L02 cells treated with alcohol. (**a**) Chemical structure and formula of GOS. (**b**) Effects of different concentrations of GOS on cell viability (*n* = 6, means ± SD). (**c**) Effects of different concentrations of ethanol on cell viability (*n* = 6, means ± SD). (**d**) Effects of GOS on the viability of L02 cells treated with ethanol (*n* = 6, mean ± SD). (**e**) The effect of GOS on the growth status of L02 cells treated with alcohol. Notes: Compared with the control (CON) group, ### *p* < 0.001; compared with the alcohol model control (MOD) group, * *p* < 0.05, ** *p* < 0.01, and *** *p* < 0.001.

Galacto-oligosaccharide (GOS) is structurally linked with one to seven galactose groups on galactose or glucose molecules [9]. The chemical structure is Gal-(Gal)*_n_*-Glc/Gal (*n* = 0–6) [10], as shown in Figure 1a. The physical and chemical properties of GOS are stable [11,12]. GOS is a white crystalline powder in appearance, soluble in water, has high moisture retention, is similar in viscosity to high fructose syrup, and has strong acid and heat resistance properties. It consists of natural functional oligosaccharides derived from animal milk with innate beneficial properties. GOS is digested by the host and exerts beneficial effects through selective metabolism in the intestine. It is enriched in breast milk and is an important prebiotic that very effectively regulates the intestinal microbiota, enhances resistance, and improves metabolic syndrome, among other properties. GOS also enhances memory, promotes the absorption of many minerals by the human body, and improves the utilization of protein in humans. It is used in food, condiments, health care products, medicine, and other products, and because of its many uses, its market prospects are very broad [13,14]. GOS has been reported to control blood flow and tissue lipid levels by inhibiting the expression of adipogenic genes and enzymes. It can also suppress systemic inflammation and reduce oxidative stress [15]. Ghosh et al. [16]. found that the intestinal barrier function in mice treated with GOS was enhanced, and the numbers of circulating macrophages and neutrophils were reduced, along with systemic inflammation. These outcomes were also reported in the study by Serino M et al. [17].

Oxidative stress, inflammation, and lipid accumulation in the liver are the current important pathological mechanisms of ALD. Because GOS exhibits effective lipid-lowering, antioxidant, and anti-inflammatory functions, no research or report on GOS in ALD has been published to date. This article first shows the protective effect of GOS on acute alcohol-induced injury in vivo and in vitro and then presents its mechanism, providing an experimental basis and theoretical support for the development of GOS as a health food with anti-ALD functions.

## 2. Materials and Methods

### 2.1. Reagents

King-Prebiotics^®^ (New Taibei City, Taiwan) 100% pure GOS was provided by New Jinshan Biotechnology Co., Ltd. (Guangdong, China). GOS consists of 0.8% disaccharides, 48% trisaccharides, 30% tetrasaccharides, 20% pentasaccharides, and 1.0% hexasaccharides. Anhydrous ethanol (analytical purity) was purchased from Aladdin Biochemical Technology Co., Ltd. (Shanghai, China).

### 2.2. Cell Culture

Human normal liver L02 cells were obtained from the Cell Bank of the Chinese Academy of Sciences. Using RPMI 1640 medium (Gibco, Waltham, MA, USA) containing 10% foetal bovine serum (HyClone, UT, USA) and 1% penicillin streptomycin (HyClone, UT, USA), normal L02 human hepatocytes were cultured in vitro with 5% CO_2_ at 37 °C. Upon reaching 80% confluence, adherent cells were subcultured or frozen.

The Cell Counting Kit-8 (CCK-8) method was used to determine the appropriate concentration of GOS and the optimal concentration of alcohol needed to establish hepatocyte injury models. With a survival rate greater than 98% as the standard, the final low, medium, and high GOS concentrations were 10 mg/mL, 20 mg/mL, and 40 mg/mL, respectively (Figure 1b). The selection of the modelling concentration was based on the half-maximal inhibitory concentration (IC50). When the alcohol concentration was 350 mM, the cell survival rate was 51 ± 13%, and the lethality rate was approximately 50%. Therefore, 350 mM was selected as the alcohol concentration for establishing the model in this experiment (Figure 1c).

### 2.3. Determination of L02 Cell Damage and the Oxidation–Antioxidant Index

Five groups were established: a control group (CON), alcohol model control group (MOD), and GOS low (GOS L), GOS medium (GOS M), and GOS high (GOS H) administration groups, with each group seeded in 6-well plates. In the GOS L, GOS M, and GOS H groups, the GOS doses were 10, 20, and 40 mg/mL, respectively. Each GOS administration group was treated with 200μL of medium containing different concentrations of GOS in 96-well plates and incubated for 12 h, and then, alcohol was added to the GOS-containing medium for a final alcohol concentration of 350 mM. The CON group was treated with the same amount of medium as the experimental groups, 200 μL of 350 mM alcohol were added to the MOD group, and the culture was maintained for another 12 h. The cell culture media were collected from each group, and the aspartate aminotransferase (AST) and alanine aminotransferase (ALT) levels in the supernatant were determined after centrifugation. After aspiration of the cell culture medium, the cells were digested with trypsin and collected, and the levels of malondialdehyde (MDA), glutathione (GSH), superoxide dismutase (SOD), catalase (CAT), glutathione peroxidase (GSH-Px), and total antioxidant capacity (T-AOC) in the cell homogenate were measured. The kits used to detect the activity of the aforementioned indicators were purchased from Nanjing Jiancheng Bioengineering Institute (Jiangsu, China), except for the MDA kit, which was purchased from Beijing Solarbio Technology Co., Ltd. (Beijing, China).

### 2.4. Animal Grouping and Administration

Fifty specific-pathogen-free (SPF) male Kunming mice weighing 18–22 g (animal production license number SCXK (Guangdong) 2018-0002) were purchased from Guangdong Medical Laboratory Animal Center (Guangzhou, Guangdong, China). The experimental technology and research design were approved and reviewed by the Institutional Review Committee of the Guangdong Medical Laboratory Animal Center (SCXK (Foshan, Guangdong, China) 2018-0002). This study was approved by the Laboratory Animal Ethics Committee of Guangdong Pharmaceutical University and was performed in strict accordance with the requirements of the “Guidelines for the Ethical Review of Laboratory Animal Welfare” (GB/T35892-2018). All procedures and animal handling followed the international guidelines for the care and use of laboratory research animals and complied with the regulations of the Guangdong Medical Laboratory Animal Center (No. 44007200065894). After adaptive feeding for 3 days in an SPF environment, the test mice were randomly allocated to 5 test groups: the CON group, MOD group, and the GOS L, GOS M, and GOS H groups, each with 10 mice (*n* = 10 animals per group). The mice in each group were numbered and weighed. The temperature of the animal breeding environment was maintained at 24.0 ± 2.0 °C, the relative humidity was 54–65%, the air was exchanged >15 times/h, and the lighting cycle was 12 h of light/12 h of dark. The laboratory animal use license number was SYXK (Guangdong) 2017-0125.

At present, the maximum tolerated intake of dietary fiber in humans has not been set, GOS has been approved as a food supplement, and its maximum dose should not exceed 16.2 g/day and up to 58 g/d when added to an authorized food category [18]. Clinical studies have also shown that at least 12 g of GOS/d can reduce food intake and appetite in overweight adults, and 18 g of GOS/d promoted better weight loss and anti-inflammatory effects; no adverse effects, such as abdominal pain, were reported by subjects throughout the study [19]. The setting of dose groups was based on the “Technical Standards for Testing & Assessment of Health Food” issued by the Ministry of Health of the People’s Republic of China, as well as our previous experiments and published papers [20,21], such as for GOS L (833.5 mg/kg body weight (BW)), GOS M (1667.0 mg/kg BW), and GOS H (3334.0 mg/kg BW).

The GOS sample solution that was prepared with double distilled water was administered by gavage to the GOS group, and the CON and MOD groups were administered double distilled water instead of GOS by gavage for 30 consecutive days. The weight of the mice was recorded weekly, and the amount of fluid administered by gavage was adjusted according to the changes in the weight of the mice. After intragastric administration on the 30th day, the GOS treatment groups and MOD group were intragastrically administered a one-time dose of 50% ethanol at 5.5 g/kg BW to induce liver injury. The establishment of the mouse model and alcohol concentration were determined according to the “Technical Standards for Testing & Assessment of Health Food” issued by the Ministry of Health of the People’s Republic of China and a previously published study [22]. The CON group was administered a corresponding volume of double distilled water, and then, the test animals were fasted for 16 h. Blood samples, liver samples, and other samples were collected and then stored at −80 °C.

### 2.5. Serum Biochemical Analysis

The collected blood was added to 2.0 mL sterile Eppendorf (EP) tubes and allowed to stand for 1 h and then centrifuged at 4 °C (3000× *g* rpm, 15 min) to separate the serum. The serum on the top of the sample was removed, and the levels of ALT, AST, alkaline phosphatase (ALP), lactate dehydrogenase (LDH), interleukin-1β (IL-1β), tumor necrosis factor-α (TNF-α), IL-6, triglycerides (TG), total cholesterol (TC), and very low-density lipoprotein (VLDL) were determined using the appropriate kits. TNF-α, IL-6, and IL-1β ELISA kits were purchased from Jiangsu Meimian Industrial Co., Ltd. (Yancheng, China). Other kits were obtained from Nanjing Jiancheng Bioengineering Institute (Nanjing, China).

### 2.6. Detection of Liver Oxidation and Antioxidant Indexes

The levels of reactive oxygen species (ROS), GSH, GSH-Px, SOD, CAT, and T-AOC were measured using the corresponding kits from Nanjing Jiancheng Bioengineering Institute. (Nanjing, China); MDA levels were measured using kits from Beijing Solarbio Technology Co., Ltd. (Beijing, China).

### 2.7. Histopathological Observation of the Liver

Approximately 0.5 cm^3^ of liver tissue was processed using standard procedures. Sections were cut to 5 µm thickness using a paraffin slicer and were stained with hematoxylin and eosin (Leagene Biotechnology, Beijing, China). Additionally, with ice cut at −20 °C, sections of 5 µm thickness were stained with oil red O (Sigma, St. Louis, MO, USA). Finally, the sections were photographed and observed in the bright field using an Olympus BX53 (Olympus, Tokyo, Japan) upright microscope for histopathology.

### 2.8. Western Blot Analysis

L02 hepatocytes and liver tissue were homogenized at a low temperature with RIPA lysis buffer (Meilun, Dalian, China) containing 1% PMSF, placed on ice for 15 min for complete lysis, and then centrifuged at 12,000× *g* rpm at 4 °C for 20 min. The bicinchoninic acid method was used for protein quantification, and the liver homogenate was then diluted; 1/4 of the sample volume of 5× sample loading buffer was added to the homogenate, and the sample was placed in a 98 °C metal bath for 10 min for denaturation. A total of 20 μg of protein sample was added to each lane of the gel, and after electrophoresis, the proteins were transferred to a polyvinylidene difluoride (PVDF) membrane (Merck KGaA, Darmstadt, Germany), which was washed 3 times with 1× TBST. The antibodies used in the experiments were purchased from Abcam (Cambridge, UK), CYP2E1 (ab28146), β-Actin (ab8227), Keap1 (ab227828), HO-1 (ab13243), Nrf2 (ab92946), NF-kB p65 (ab16502), p-NF-kB p65 (ab76302), c-Jun N-terminal kinase (JNK) (ab179461), p-JNK (ab76572), p38 MAPK (ab170099), and p-p38 MAPK (ab195049). After adding the primary antibody and incubating it with the membrane for 12 h at 4 °C, the secondary antibody was added and incubated for 1 h. The PVDF membrane was washed 2–3 times, and an HRP-enhanced chemiluminescence (ECL) reagent (Meilun, Dalian, China) was added to develop the blot, which was photographed. Data analysis was performed with an automatic gel imager (ChemiDoc XRS+, Bio-Rad, Hercules, CA, USA).

### 2.9. RT‒PCR Analysis

Total RNA extraction reagent RNAiso Plus reagent (Takara, Dalian, China) was added to L02 cells and liver tissue to extract total RNA. The total RNA content and purity were determined. The DNA was removed with a kit (Takara, Dalian, China), and the RNA was reverse transcribed into cDNAs. The DNA template used was 2 μg. The target gene primer sequences (Sangon Biotech (Shanghai) Co., Ltd., Shanghai, China) are shown in Table 1 and Table 2. The PCR program was as follows: 95 °C for 3 min, followed by 40 cycles of 95 °C for 5 s and 60 °C for 30 s. β-actin was used as the internal reference gene, and after the reaction was complete, the 2^−ΔΔCt^ method was used to analyze the gene expression in the groups and calculate the expression of the detected genes relative to β-actin.

### 2.10. Statistical Analysis

The data obtained in this experiment were analyzed and processed using SPSS 20.0 (IBM^®^, Armonk, NY, USA) and GraphPad Prism 8.0 software (GraphPad Software, San Diego, CA, USA). One-way ANOVA was used for comparisons between more than two groups, and a *t*-test was used for comparisons between the two groups. A *p* value less than 0.05 indicated statistical significance, and the final results of the experiment are presented as the means ± SD.

## 3. Results

### 3.1. Protective Effects of GOS on Alcohol-Induced Injury in L02 Liver Cells

L02 hepatocytes were administered the GOS intervention for 12 h and then treated with alcohol for 12 h. The cell survival rate was measured by the CCK-8 method to detect the protective effect of GOS on alcohol-induced L02 hepatocyte damage. Compared to the alcohol model group, the GOS intervention groups significantly increased the survival rate of liver cells and inhibited liver cell damage, as shown in Figure 1d. In addition, as shown in Figure 1e, compared to the CON group, the number of normal liver cells (grey and dark) in the MOD group was noticeably reduced, the number of dead cells (bright) was prominently increased, and the morphology of many living cells was changed, showing a long-horned state. In the GOS H group, the number of normal liver cells (grey and dark) was significantly increased, the cell morphology was relatively normal, and the number of dead cells (bright) was noticeably reduced.

**Figure 2:**
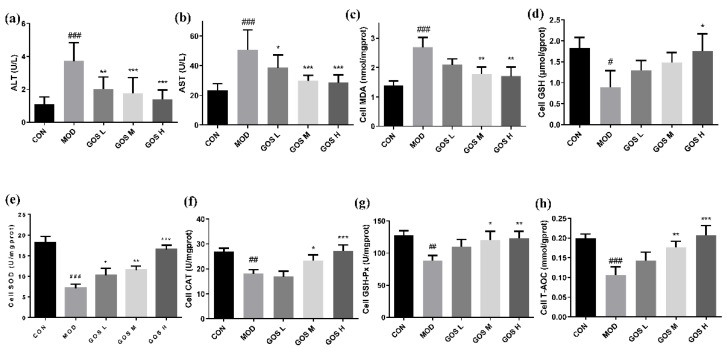
Effect of GOS on alcohol-induced injury, oxidative damage index, and antioxidant enzyme index in L02 liver cells (*n* = 6, means ± SD). (**a**) ALT. (**b**) AST. (**c**) Cell MDA. (**d**) Cell GSH. (**e**) Cell SOD. (**f**) Cell CAT. (**g**) Cell GSH-Px. (**h**) Cell T-AOC. Notes: Compared with the MOD group, * *p* < 0.05, ** *p* < 0.01, and *** *p* < 0.001; compared with the CON group, # *p* < 0.05, ## *p* < 0.01, and ### *p* < 0.001.

The culture medium supernatant was collected, and ALT and AST activities were measured. Compared to the MOD group, the GOS intervention groups prominently reduced ALT and AST activities in the culture medium, as shown in Figure 2a,b. Based on these results, GOS protects against liver cell damage induced by alcohol.

### 3.2. Effects of GOS on the Oxidative Damage Indexes of L02 Liver Cells

The MDA content in the MOD group was noticeably increased, and the L02 cells underwent lipid peroxidation upon alcohol exposure. Compared to the MOD group, the MDA content in the L02 cells from the GOS M and GOS H groups was significantly decreased, as shown in Figure 2c. Compared to the MOD group, the GSH content in the L02 cells from the GOS H group was prominently increased, as shown in Figure 2d. Thus, the GOS intervention increases the GSH content and reduces alcohol-induced oxidative damage in L02 liver cells.

### 3.3. Effects of GOS on the L02 Liver Cell Antioxidant Enzyme Index

The levels of SOD, CAT, GSH-Px and T-AOC in the cells were detected. The results are shown in Figure 2e–h. Compared to the CON group, the levels of SOD, CAT, GSH-Px, and T-AOC in the MOD group were prominently reduced. Compared with the MOD group, each GOS-treated L02 cell group showed a significantly increased SOD level, which was increased in a dose-dependent manner, as shown in Figure 2e; in the GOS M and GOS H groups of L02 cells, the CAT, GSH-Px, and T-AOC levels were significantly increased, as shown in Figure 2f–h. These results show that GOS strengthens the activities of antioxidant enzymes and improves the antioxidant capacity of L02 liver cells.

### 3.4. Effects of GOS on the Body Weight and Liver of Mice

**Figure 3:**
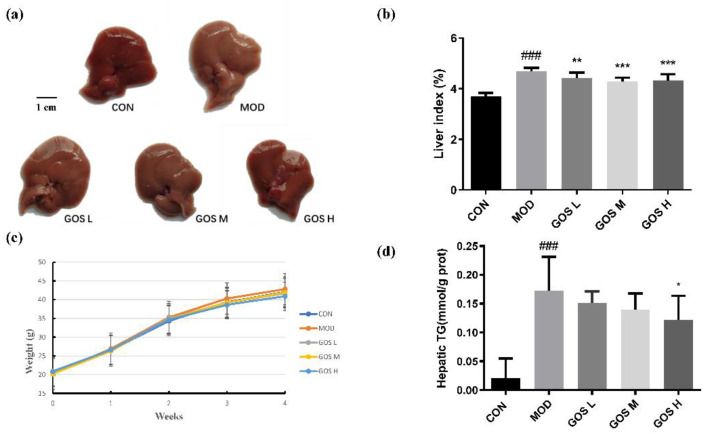
Liver appearance, liver index, weight change graph, and liver TG content for each group. (**a**) The appearance of the livers of mice in each group. (**b**) The effect of GOS on the liver index in mice with alcohol-induced liver injury (*n* = 10, means ± SD). (**c**) The effect of GOS on mouse BW (*n* = 10, means ± SD). (**d**) Hepatic TG (*n* = 10, means ± SD). Notes: Compared with the MOD group, * *p* < 0.05, ** *p* < 0.01, and *** *p* < 0.001; compared with the CON group, ### *p* < 0.001.

The appearance of the livers of mice in each group is shown in Figure 3a. The liver of the MOD group was whitish, swollen, and had a rough surface with obvious granularity; the GOS-treated groups had healthier livers that tended to be normal in size without obvious granularity, and the liver tissue of the GOS H group was similar to that of the CON group. The ratio of the weight of the liver to BW is called the liver index; the higher the value, the more serious the liver injury. The liver index was significantly increased in the MOD group compared to the CON group. All GOS-treated groups had a significantly reduced liver index and attenuated liver enlargement due to alcohol exposure (Figure 3b). No significant difference in BW was observed between groups during the experiment (*p* > 0.05), indicating that GOS had no significant effect on the BW of the mice (Figure 3c). In our measurements of liver TG levels, we found that the MOD group had significantly higher liver TG levels than the CON group, the GOS administration group reduced TG levels, and the GOS H group significantly reduced liver TG levels (Figure 3d).

**Figure 4:**
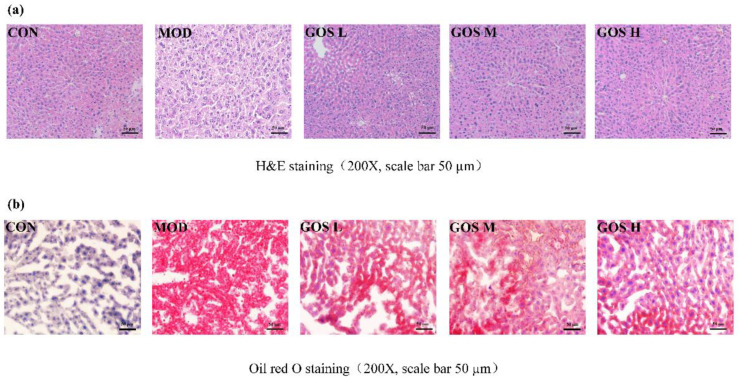
Histopathological observation of the liver. (**a**) Pathological image of liver tissue stained with haematoxylin and eosin (H&E) (200×, scale bar = 50 μm). (**b**) Pathological image of liver tissue stained with oil red O (200×, scale bar = 50 μm).

H&E staining was performed on liver sections. Fat was dissolved in a fat-soluble reagent, and vacuoles of varying sizes were observed in the locations where the fat had been eliminated. The pathological analysis of the slices showed that the liver cells in the MOD group were arranged in a disordered manner, the boundaries between cells were blurred, and fat-replacing vacuoles of different sizes were observed in the cells. Fatty degeneration was evident; the degree of steatosis in the GOS L group was similar to that of the MOD group, and no significant improvement was observed over time. The liver cell structure and liver cord arrangement in the GOS M and GOS H groups were basically close to normal. No fat vacuoles appeared, and liver pathological conditions were significantly attenuated, as shown in Figure 4a. The results of oil red O staining showed that there were no obvious red lipid droplets in the liver of the CON group and a large accumulation of red lipid droplets in the liver of the MOD group, and the GOS administration group could reduce the number and area of lipid droplets in the liver (Figure 4b). These results suggest that GOS attenuates hepatic steatosis caused by alcoholic liver injury.

### 3.5. Effects of GOS on Serum Levels of Proteins Related to Liver Function in Mice

**Figure 5:**
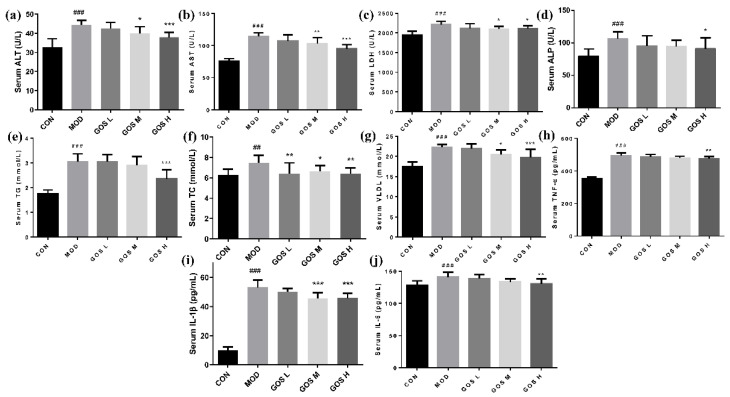
Effects of GOS on serum levels of proteins related to liver function and inflammatory factors in mice (*n* = 10, means ± SD). (**a**) Serum ALT. (**b**) Serum AST. (**c**) Serum LDH. (**d**) Serum ALP. (**e**) Serum TG. (**f**) Serum TC. (**g**) Serum VLDL. (**h**) Serum TNF-α. (**i**) Serum IL-1β. (**j**) Serum IL-6. Notes: Compared with the MOD group, * *p* < 0.05, ** *p* < 0.01, and *** *p* < 0.001; compared with the CON group, ## *p* < 0.01, and ### *p* < 0.001.

Compared with the CON group, the levels of ALT, AST, ALP, and LDH in the MOD group were noticeably increased. Compared to the MOD group, the GOS M and H administration groups showed prominent decreases in the alcohol-induced increases in serum ALT and AST and LDH levels, and the effect was dose-dependent, as shown in Figure 5a–c. The GOS H group showed significantly reduced ALP activity, as shown in Figure 5d. Alcohol intake leads to impairment in the tricarboxylic acid cycle and fatty acid oxidation. By measuring the serum lipid levels, we found that the serum TG, TC, and VLDL levels in the MOD group were considerably higher than those in the CON group, as shown in Figure 5e–g. Compared to the MOD group, the GOS H dose group showed a significantly reduced serum TG content; all GOS dose groups showed significantly reduced serum TC levels. GOS dose dependently reduced serum VLDL levels, with the GOS M and H groups showing significant effects. Based on these results, GOS alleviates the dysfunction of lipid metabolism caused by alcohol.

### 3.6. Effects of GOS on Serum Levels of Inflammatory Factors in Mice

TNF-α, IL-1β, and IL-6 are crucial cytokines involved in the occurrence and development of liver inflammation. Serum levels of these cytokines increase significantly when cells are damaged and inflammation develops. As shown in Figure 5h–j, the MOD group exhibited prominently increased serum levels of TNF-α, IL-6, and IL-1β, indicating that the mice had inflammatory cell infiltration. Compared to the MOD group, the GOS H group showed a significantly reduced serum TNF-α level after alcohol administration; the GOS M and GOS H groups showed a significantly reduced IL-1β level, and the effect was extremely significant; and the GOS H group showed significantly reduced serum IL-6 levels. Thus, GOS attenuates alcohol-induced inflammation.

### 3.7. Effects of GOS on the Oxidative Damage Indexes in the Mouse Liver

**Figure 6:**
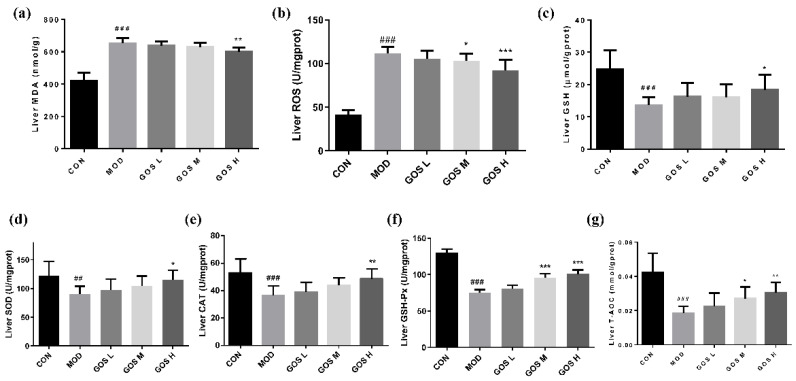
Effects of GOS on the oxidative damage indexes and the activity of antioxidant enzymes in mouse liver (*n* = 10, means ± SD). (**a**) Liver MDA. (**b**) Liver ROS. (**c**) Liver GSH. (**d**) Liver SOD. (**e**) Liver CAT. (**f**) Liver GSH-Px. (**g**) Liver T-AOC. Notes: Compared with the MOD group, * *p* < 0.05, ** *p* < 0.01, and *** *p* < 0.001; compared with the CON group, ## *p* < 0.01, and ### *p* < 0.001.

Elevated MDA and ROS levels are major signs of oxidative damage in the liver, and their content may reflect the degree of liver lipid peroxidation. The levels of MDA and ROS in mouse livers were tested to determine whether GOS reduced the degree of oxidative stress in the liver, and the results are shown in Figure 6. Compared to the CON group, the MDA and ROS contents in the MOD group were noticeably increased, indicating that alcohol caused oxidative damage to the liver of the MOD group, as shown in Figure 6a,b. Compared to the MOD group, the ROS level in the GOS M group was significantly reduced, and the hepatic MDA and ROS contents were prominently reduced in the GOS H group, indicating that GOS reduces oxidative damage in the liver.

GSH has strong reducing properties and plays an important role in maintaining the oxidation–antioxidant balance in the body [23,24]. A large amount of ROS is produced in the process of alcohol metabolism to deplete GSH. The GSH level may indirectly reflect the redox state of the body. To determine whether GOS can alleviate the oxidation level in the body, The GSH content in the mouse liver was measured to determine whether GOS alleviated oxidation in the body, and the results are shown in Figure 6c. Compared to the CON group, the hepatic GSH content was prominently lower in the MOD group, indicating that alcohol exposure leads to the metabolism of the reducer GSH. Compared to the MOD group, the hepatic GSH level in the GOS H group was significantly increased, indicating that GOS alleviates liver oxidation.

### 3.8. Effects of GOS on the Activity of Antioxidant Enzymes in Mouse Liver

The aforementioned research results show that GOS reduce the level of lipid oxidation in the liver and enhance the oxidation–antioxidant balance. Therefore, the activities of the main antioxidant enzymes SOD, GSH-Px, CAT, and T-AOC were measured to determine the effect of GOS on the antioxidant level in the liver. SOD scavenges superoxide anion free radicals and reduces oxidative damage in the body. Compared to the MOD group, SOD activity in the livers in the GOS H group was noticeably increased, as shown in Figure 6d. Compared to the MOD group, CAT activity in the livers of mice in the GOS H group was substantially increased, as shown in Figure 6e. GSH-Px specifically catalyzes the reaction between GSH and ROS and reduces the ROS level in the body, thereby protecting the body from ROS-induced damage and promoting normal cellular function. Compared to that in the MOD group, GSH-Px activity in the GOS M and GOS H groups was prominently increased in a dose-dependent manner, as shown in Figure 6f. The T-AOC of the liver, which was reflected in the T-AOC test result, is shown in Figure 6g. Compared to the MOD group, the hepatic T-AOC of the mice in the GOS M and GOS H groups increased significantly and showed a dose-dependent increasing trend. These results indicate that GOS strengthen the antioxidant capacity of the liver by increasing the activities of antioxidant enzymes.

### 3.9. Effects of GOS on the Alcohol Metabolism Enzyme CYP2E1

**Figure 7:**
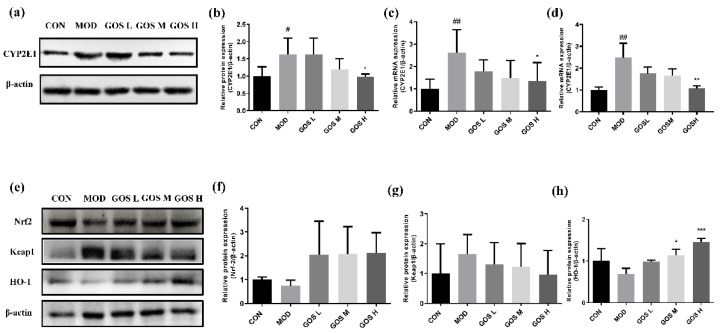
Effects of GOS on the CYP2E1 and Keap1/Nrf2/HO-1 pathway. (**a**) Western blot images of CYP2E1 in mouse liver (*n* = 3, means ± SD). (**b**) Protein expression levels of CYP2E1 in mouse liver (*n* = 3, means ± SD). (**c**) CYP2E1 mRNA expression in mouse liver (*n* = 6, means ± SD). (**d**) CYP2E1 mRNA expression in L02 liver cells (*n* = 6, means ± SD). (**e**) Western blot images of the Keap1/Nrf2/HO-1 pathway in mouse liver (*n* = 3, means ± SD). (**f**) Protein expression levels of Nrf2 in mouse liver (*n* = 3, means ± SD). (**g**) Protein expression levels of Keap1 in mouse liver (*n* = 3, means ± SD). (**h**) Protein expression levels of HO-1 in mouse liver (*n* = 3, means ± SD). Notes: Compared with the MOD group, * *p* < 0.05, ** *p* < 0.01, and *** *p* < 0.001; compared with the CON group, # *p* < 0.05, ## *p* < 0.01.

CYP2E1 activity is significantly increased during heavy drinking. It participates in the oxidative metabolism of alcohol and produces many ROS, which leads to oxidative damage in the liver [25]. Therefore, a Western blot experiment was performed to detect and analyze CYP2E1 protein expression in mouse livers, as shown in Figure 7a,b. Compared to the CON group, CYP2E1 protein expression in the MOD control group was increased. Compared to the MOD group, the expression of the CYP2E1 protein in the GOS administration groups was decreased.

CYP2E1 mRNA expression was quantified and analyzed in mouse liver **(**Figure 7c) and L02 cells (Figure 7d) using RT‒PCR, and the results showed increased expression of the CYP2E1 mRNA in the MOD group. As the dose of GOS increased, the expression of CYP2E1 mRNA gradually decreased, and the GOS H group showed a significant decrease in its expression.

### 3.10. Effects of GOS on the Antioxidant Keap1/Nrf2/HO-1 Pathway

Under normal circumstances, Keap1 binds Nrf2 in the cytoplasm; therefore, Nrf2 is typically maintained in the inactive state. In response to oxidative stress, however, Nrf2 dissociates from Keap1, becomes active, and then enters the nucleus to initiate the expression of the antioxidant gene HO-1.

Previous experiments have indicated that GOS increase the antioxidant properties of the mouse liver. In this experiment, Western blotting was performed to detect the expression of the Keap1, Nrf2, and HO-1 proteins and clarify the antioxidant and hepatoprotective mechanism of GOS. The results are shown in Figure 7e. Compared to the MOD group, GOS administration increased Nrf2 protein expression (Figure 7f), and the GOS M and GOS H groups exhibited prominently increased expression of the HO-1 protein (Figure 7h). GOS administration reduced Keap1 protein expression, as shown in Figure 7g. Therefore, GOS activates the antioxidant Keap1/Nrf2/HO-1 pathway to reduce liver damage caused by oxidative stress.

### 3.11. Effects of GOS on the MAPK/NF-κB Pathways

**Figure 8:**
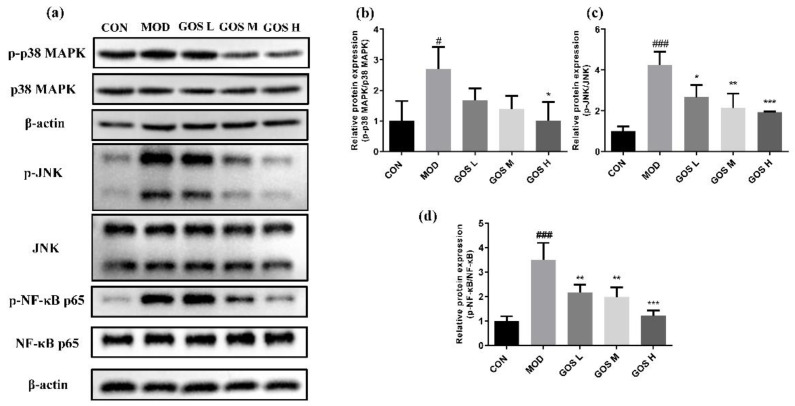
Effects of GOS on the NF-κB and MAPK pathways in L02 liver cells. (**a**) Western blot images of the NF-κB and MAPK pathways in L02 liver cells (*n* = 3, means ± SD). (**b**) Phosphorylated protein expression levels of p38 MAPK (*n* = 3, means ± SD). (**c**) Phosphorylated protein expression levels of JNK (*n* = 3, means ± SD). (**d**) Phosphorylated protein expression levels of NF-κB p65 (*n* = 3, means ± SD). Notes: Compared with the MOD group, * *p* < 0.05, ** *p* < 0.01, and *** *p* < 0.001; compared with the CON group, # *p* < 0.05, ### *p* < 0.001.

**Figure 9:**
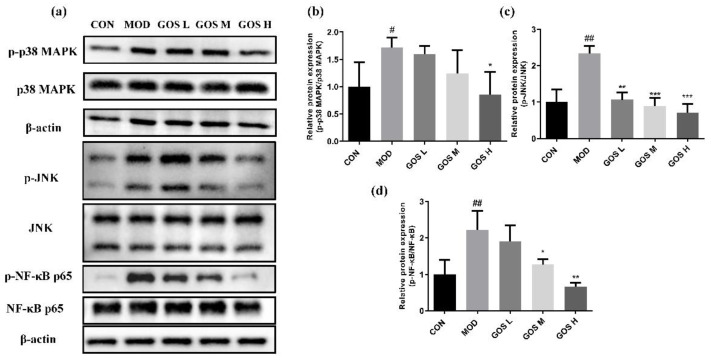
Effects of GOS on the NF-κB and MAPK pathways in mouse liver. (**a**) Western blot images of the NF-κB and MAPK pathways in mouse liver (*n* = 3, means ± SD). (**b**) Phosphorylated protein expression levels of p38 MAPK (*n* = 3, means ± SD). (**c**) Phosphorylated protein expression levels of JNK (*n* = 3, means ± SD). (**d**) Phosphorylated protein expression levels of NF-κB p65 (*n* = 3, means ± SD). Notes: Compared with the MOD group, * *p* < 0.05, ** *p* < 0.01, and *** *p* < 0.001; compared with the CON group, # *p* < 0.05, ## *p* < 0.01.

Overactivated NF-κB and MAPK pathways also promote liver inflammation and exert important effects on cell inflammation and apoptosis [26,27]. In this experiment, Western blot experiments were performed to detect the levels of phosphorylated p38 MAPK, JNK, and NF-κB in L02 cells (Figure 8) and mouse livers (Figure 9) to determine the protective effect of GOS on alcohol-induced injury. Compared to the CON group, the MOD group displayed prominently increased levels of the phosphorylated p38 MAPK, JNK, and NF-κB proteins. Compared to the MOD group, the levels of phosphorylated p38 MAPK, JNK, and NF-κB proteins in L02 cells from the GOS H group were significantly reduced (Figure 8). Compared to the MOD group, the GOS H group showed noticeably reduced levels of phosphorylated p38 MAPK and NF-κB proteins in the mouse liver (Figure 9). Compared to the MOD group, GOS noticeably reduced the levels of phosphorylated JNK protein in the mouse liver in a dose-dependent manner (Figure 9). Thus, the activities of JNK, p38 MAPK, and NF-κB are inhibited, indicating that GOS inhibits the oxidative and inflammatory pathways in the body.

### 3.12. Effects of GOS on the NOD-like Receptor Family Pyrin Domain Containing 3 (NLRP3) Inflammasome and Inflammatory Genes

Excessive activation of NLRP3 inflammasomes releases a large amount of the inflammatory factors IL-18 and IL-1β, leading to aggravation of the systemic inflammatory response and ultimately leading to pyrolysis. The NLRP3 inflammasome is also activated by NF-κB. Previous experimental results showed NF-κB overactivation after exposure to alcohol, and GOS administration significantly reduced the activity of NF-κB. Therefore, the activity of the NLRP3 inflammasome was subsequently tested to determine whether GOS affected the NLRP3 inflammasome.

**Figure 10:**
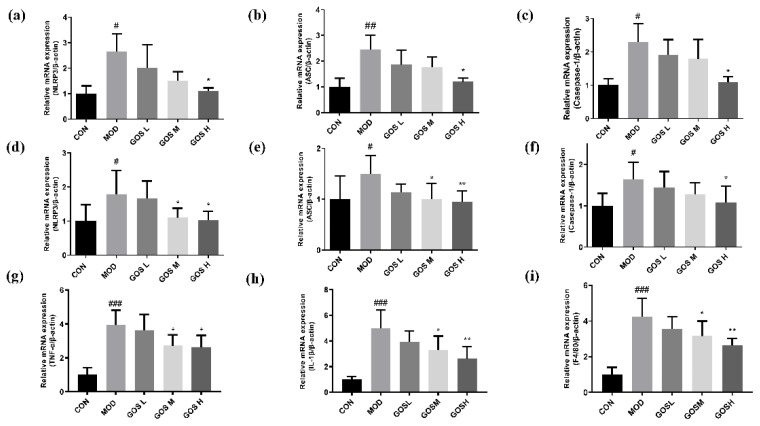
Effects of GOS on the NLRP3 inflammasome and inflammatory genes (*n* = 6, means ± SD)**.** (**a**–**c**) NLRP3, ASC, and Caspase-1 mRNA expression in L02 liver cells. (**d**–**f**) NLRP3, ASC and Caspase-1 mRNA expression in mouse livers. (**g**–**i**) TNFα, IL-1β and F4/80 mRNA expression in mouse livers. Notes: Compared with the MOD group, * *p* < 0.05, ** *p* < 0.01; compared with the CON group, # *p* < 0.05, ## *p* < 0.01, and ### *p* < 0.001.

Quantitative analyses of NLRP3, ASC, and Caspase-1 mRNA expression were performed using RT‒PCR. The results are shown in Figure 10. Compared to the CON group, the expression of the NLRP3, ASC, and Caspase-1 mRNAs was significantly increased in L02 cells from the alcohol-exposed MOD group, as shown in Figure 10a–c, and mouse liver, as shown in Figure 10d–f. Compared to the MOD group, the GOS H group prominently reduced the expression of the NLRP3, ASC, and Caspase-1 mRNAs in L02 cells, as shown in Figure 10a–c. Compared with the MOD group, the GOS M and GOS H treatments reduced the expression of the NLRP3, ASC, and Caspase-1 mRNAs in the mouse liver in a dose-dependent manner, as shown in Figure 10d–f.

In addition, we examined the expression of inflammatory genes, including TNFα and IL-1β, in the mouse liver. As shown in Figure 10g,h, GOS can significantly inhibit the expression of inflammatory genes. We also examined the expression of the macrophage cell marker F4/80. In the course of alcoholic liver injury, macrophages are the main cause of inflammatory infiltration [28]. The results showed that the GOS administration group also reduced the expression of F4/80 (Figure 10i). These results indicated that GOS reduces the maturation and release of proinflammatory cytokines and alleviates inflammatory damage to the liver.

## 4. Discussion

In this study, we first assessed the hepatoprotective effect of GOS on alcohol-induced liver damage and found that GOS effectively reduces liver damage caused by exposure to a large volume of alcohol in vivo and in vitro. This study provides an experimental basis for research on the protective effects of GOS on ALD.

The GOS intervention prominently reduced the levels of AST and ALT in the cell culture medium supernatant, the levels of ALT, AST, ALP, LDH, and the inflammatory factors TNF-α, IL-6, and IL-1β in the serum of mice, and the levels of F4/80,TNF-α, and IL-1β in the liver, indicating that the GOS intervention prevents the damaging effects of alcohol consumption. Levels of the lipids TG, TC, and VLDL in mouse blood samples and the TG content in the liver of the mice also significantly decreased after the GOS intervention. Combined with the histopathological assessment of the liver slices, the steatosis in the GOS intervention groups was significantly reduced, indicating that GOS attenuates alcohol-induced lipid metabolism dysfunction and elevated blood lipid levels.

The MDA and ROS contents in the MOD group increased significantly, indicating that the liver underwent alcohol-induced oxidative damage. The GOS intervention significantly reduced the levels of MDA and ROS, indicating that GOS reduced the oxidative damage in the liver caused by alcohol exposure. In addition, GSH levels in cells and liver tissues were measured and negatively correlated with the MDA content, indicating that a large amount of GSH was metabolized after alcohol intake. The GSH level in the GOS intervention groups was significantly increased, indicating that GOS alleviates the oxidative damage caused by alcohol. In addition, the GOS intervention prominently increased the activities of the antioxidant enzymes SOD, CAT, and GSH-Px, including elevated T-AOC levels, indicating that GOS strengthens the antioxidant capacity of cells and the liver through their ability to reduce the oxidative damage caused by alcohol exposure.

ALD is associated with increased CYP2E1 activity [29]. CYP2E1 is a key factor contributing to alcohol-induced inflammation and oxidative stress and may increase the toxic effects of alcohol [30]. Studies of mice lacking the CYP2E1 gene have proven that CYP2E1 plays important roles in oxidative stress, intestinal leakage, and endotoxaemia and is involved in hepatocyte apoptosis and steatohepatitis [31,32]. Numerous experimental studies have shown that after alcohol treatment, CYP2E1 catalytic activity and protein expression increase significantly [33,34,35]. Similarly, in the present study, CYP2E1 protein and mRNA expression increased in the MOD group. The GOS intervention inhibited the expression of the CYP2E1 protein and mRNA, thereby reducing alcohol metabolism by the CYP2E1 pathway and attenuating hepatic oxidative damage.

Nrf2 is a key factor involved in redox and metabolic regulation in the body [36]. Nrf2 and its negative regulator Keap1 constitute a defense system against oxidative-stress-induced damage [37]. Generally, Keap1 binds to Nrf2 in the cytoplasm to maintain Nrf2 at a relatively stable level. At this time, Nrf2 stably resides in the cytoplasm in an inactive state. When cells are stimulated with ROS or other stimuli, Nrf2 is activated, dissociates from Keap1, and is then transferred to the nucleus, where it binds to antioxidant response elements to induce the expression of the downstream antioxidant protein HO-1 [38]. In the present study, alcohol exposure partially activated Nrf2 to respond to the alcohol-induced oxidative stress in the body. The Keap1/Nrf2 pathway plays an extremely key role in the antioxidant response to damage and maintains the “oxidation–antioxidant system” in the body; it is also closely related to the physiological process of oxidative stress-induced damage in individuals with ALD. Here, the antioxidant Keap1/Nrf2/HO-1 pathway was studied, and GOS inhibited the expression of Keap1, increased the expression of Nrf2, and increased the expression of the downstream antioxidant factor HO-1. GOS promotes the activation of the Keap1/Nrf2 antioxidant program to improve the antioxidant capacity of the body.

Alcohol and metabolites activate the MAPK and NF-κB signaling pathways, which can lead to inflammation and apoptosis and subsequently cause substantial damage to the liver. GOS inhibited the activation of the MAPK pathway and reduced the phosphorylation of p38 MAPK and JNK. In addition, NF-κB activity was also inhibited by GOS, and its phosphorylation level in mice was significantly reduced after GOS administration. Therefore, we inferred that GOS inhibits the activation of the NF-κB and MAPK signaling pathways, thereby reducing liver inflammation and liver cell apoptosis and playing a role in preventing and treating alcoholic liver injury.

Notably, an increasing number of studies have shown that ALD is inextricably linked to the intestinal flora. Alcohol metabolism in the body causes intestinal damage, disrupts intestinal barrier permeability, and produces oxidative-stress-induced damage and inflammation in the intestine [39,40,41,42]. Meanwhile, GOS, a prebiotic, exerts a good effect on regulating and improving the intestinal flora, which has been confirmed in many studies [43,44,45]. We speculate that GOS might exert an effect on ALD by altering the intestinal environment and intestinal flora. GOS is metabolized by the microbiota in the caecum, such as short-chain fatty acids (SCFAs), a typical microbial metabolite that is an important mediator between intestinal flora and the host [46]. In a mouse NAFLD model, our research group found that GOS reduces the contents of SCFAs such as acetic acid, propionic acid, and hexanoic acid, improves the intestinal flora environment, and regulates the structure of metabolites to maintain the metabolic homeostasis of the body [20]. In the future, we will continue to explore the effects of GOS on intestinal flora and intestinal barrier proteins to provide stronger theoretical support for GOS as a functional food with anti-ALD properties.

## 5. Conclusions

**Figure 11:**
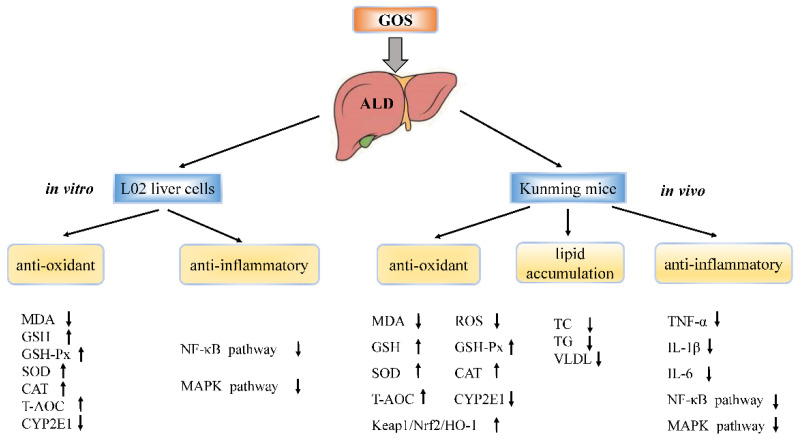
Possible mechanisms by which GOS alleviates ALD. Notes: ↑ indicates increase; ↓ indicates inhibition.

In summary, GOS inhibits CYP2E1, promotes the activation of the antioxidant Keap1/Nrf2 pathway in the liver, inhibits the activation of the MAPK/NF-κB pathways, inhibits NLRP3 inflammasome activity, enhances the antioxidant capacity of the body, and inhibits inflammation, thereby reducing oxidative stress, lipid peroxidation, inflammatory cell infiltration, and apoptosis caused by a large amount of alcohol intake and protecting against alcohol-induced liver injury (Figure 11). GOS may be a promising dietary nutrient for the prevention and mitigation of ALD in the future, and it may be used as a functional food, which has a certain advantage in the development of treatments for ALD.

## Figures and Tables

**Table 1:** Human primer sequences for RT‒PCR.

Gene Name	Primer Sequence (5′→3′)
CYP2E1	F: ACAGAGACCACCAGCACAAC
	R: TCCTTGATGGCAGGGATTCG
NLRP3	F: GCATTTCCTCTCTAGCTGTTCCT
	R: TTAGGCTTCGGTCCACACAGAAAG
ASC	F: TACCTGGAGACCTACGGCG
	R: TATAAAGTGCAGGCCCTGGTG
Caspase-1	F: ATCCGTTCCATGGGTGAAGG
	R: CGTGCTGTCAGAGGTCTTGT
β-actin	F: CATGTACGTTGCTATCCAGGC
	R: CTCCTTAATGTCACGCACGAT

**Table 2:** Mouse primer sequences for RT‒PCR.

Gene Name	Primer Sequence (5′→3′)
CYP2E1	F: CGTTGCCTTGCTTGTCTGGA
	R: AAGAAAGGAATTGGGAAAGGTCC
NLRP3	F: CCACATCTGATTGTGTTAATGGCT
	R: GGGCTTAGGTCCACACAGAA
ASC	F: CCATCCTGGACGCTCTTGAA
	R: GTGAGCTCCAAGCCATACGA
Caspase-1	F: CCGCGGTTGAATCCTTTTCAG
	R: TGTGCGCATGTTTCTTTCCC
TNF-α	F:GATCGGTCCCCAAAGGGATG
	R: CCACTTGGTGGTTTGTGAGTG
IL-1β	F: TGCCACCTTTTGACAGTGATG
	R: TGATGTGCTGCTGCGAGATT
F4/80	F: TGACTCACCTTGTGGTCCTAA
	R: CTTCCCAGAATCCAGTCTTTCC
β-actin	F: AGCAAGCAGGAGTACGATGAG
	R: GGTGTAAAACGCAGCTCAGTAA

## Data Availability

The data presented in this study are available in article.

## Abbreviations

ALD	alcoholic liver disease
GOS	galacto-oligosaccharide
CYP2E1	cytochrome P450 protein 2E1
Keap1	Kelch-like ECH-associated protein 1
Nrf2	nuclear factor erythroid-2-related factor 2
HO-1	haem oxygenase-1
FDA	Food and Drug Administration
ALT	alanine aminotransferase
AST	aspartate aminotransferase
MDA	malondialdehyde
GSH	glutathione
SOD	superoxide dismutase
CAT	catalase
GSH-Px	glutathione peroxidase
T-AOC	total antioxidant capacity
ALP	alkaline phosphatase
LDH	lactate dehydrogenase
TNF-α	tumour necrosis factor-α
IL-1β	interleukin-1β
IL-6	interleukin-6
TC	serum total cholesterol
TG	triglyceride
VLDL	very low-density lipoprotein
ROS	reactive oxygen species
MAPK	mitogen-activated protein kinase
JNK	c-Jun N-terminal kinase
NF-κB	nuclear factor kappa-B
p38 MAPK	p38 mitogen-activated protein kinase
NLRP3	NOD-like receptor family pyrin domain containing 3
SCFA	short-chain fatty acid
